# Cesarean section in Shanghai: women’s or healthcare provider’s preferences?

**DOI:** 10.1186/1471-2393-14-285

**Published:** 2014-08-22

**Authors:** Wei Deng, Reija Klemetti, Qian Long, Zhuochun Wu, Chenggang Duan, Wei-Hong Zhang, Carine Ronsmans, Yu Zhang, Elina Hemminki

**Affiliations:** School of Public Health, Fudan University, 138 Yi Xue Yuan Road, P.O. Box 250, Shanghai, 200032 China; Key Laboratory of Public Health Safety, Ministry of Education (Fudan University), Shanghai, China; National Institute for Health and Welfare, Helsinki, Finland; Global Health Research Center, Duke Kunshan University, Kunshan, China; International Centre for Reproductive Health, Ghent University, Ghent, Belgium; School of Public Health, Université libre de Bruxelles (ULB), Brussels, Belgium; London School of Hygiene and Tropical Medicine, London, UK

**Keywords:** Cesarean section, Clinical indications, Delivery mode preference, Healthcare provider’s suggestion, Urban China

## Abstract

**Background:**

Cesarean section (CS) rate has increased rapidly over the past two decades in China mainly driven by non-medical factors. This study was to compare recalled preferences for CS among first-time mothers in early and late pregnancy with actual delivery mode; to explore factors related to CS preference and CS performed without medical indications; and to consider the role of healthcare providers in delivery mode preferences.

**Methods:**

An anonymous questionnaire survey, combined with data on CS indications taken from the patient record, was conducted among 272 first-time mothers having their first postnatal check-up in one university affiliated obstetrics and gynecology hospital in Shanghai, China, between September 2006 and January 2007. Logistic regression was used to study factors related to the recalled preference for CS and CS performed without medical indication, adjusting for maternal age, education and income.

**Results:**

The CS rate was 57% (151/263) among all women, 17% with medical indications and 40% without medical indications. For women without medical indications for CS (n = 215), there was no significant difference between women’s preference for CS in early (25%) and late pregnancy (28%); 48% of women actually had CS. Women recalled preferring a vaginal delivery but who had CS were more likely to have had a CS suggested by a prenatal care doctor [OR (95% CI): 20 (3.88-107.1)] or by a delivery obstetrician [OR (95% CI): 26 (6.26-105.8)]. Among women recalled preferring and having CS, a suggestion from the prenatal care doctor to have CS was very common.

**Conclusions:**

In the primiparous women without a medical indication for CS, women recall of a provider suggestion for CS was a strong predictor of CS both among women who recalled a preference for CS and among women who recalled a preference for vaginal delivery. Public health education needs strengthening, including discussion of the risks associated with CS and psychological and social support given to women to help them prepare for and cope with childbirth.

**Electronic supplementary material:**

The online version of this article (doi:10.1186/1471-2393-14-285) contains supplementary material, which is available to authorized users.

## Background

Over the past decades, cesarean section (CS) rates have increased worldwide [1, 2]. In China, the nationwide CS rate rose from 3.4% in 1988 to 39% in 2008, with a higher rate in urban areas (64% in 2008) [3]. In Shanghai, already in 2002 over half of women gave birth by CS [4]. However, CSs are associated with increased risks of maternal and neonatal mortality and morbidity, and unnecessary CSs are a socioeconomic burden on individuals and healthcare systems [5–10].

In addition to medical reasons, such as older age of first-time mothers, high birth weight, prematurity and breech deliveries [11, 12], women’s preference for CS in the absence of medical reasons or/and supply induced this demand have contributed to the increase CS rate [13, 14]. Studies in China have reported various reasons for a maternal request for CS, including choice of a specific birth date, fear of pain, the wish to keep fit, and desire to obtain better health for the child and herself [15–20]. Given that much of the knowledge and information about childbirth is obtained during pregnancy, healthcare providers’ attitudes towards CS are likely to be an important influence on women’s views. For healthcare providers, CS is being performed at a manageable timing and relative short duration, or as a defensive medical practice for fear of malpractice accusations. It has also been argued that healthcare providers in China opt for CS partly due to profits gained from doing them [9, 19, 21].

Given a long course of pregnancy, we raise a study question whether women’s preference for delivery mode changes and what factors are associated with the change. This study investigated first-time mothers recalled preferences in early and late pregnancy regarding CS as compared to the actual delivery mode, as well as considering factors related to CS preference and CS performed without medical indications, particularly the role of healthcare providers.

## Methods

A retrospective study was conducted in one university affiliated obstetrics and gynecology hospital in Shanghai, China, from September 2006 to January 2007. It is a high-level referral hospital providing 24-hour obstetric services, with over 3000 deliveries in 2006. In this hospital, obstetricians provided a continuum maternal care including prenatal care (e.g. physical check-ups, examinations and health education on childbirth), attending vaginal or operative delivery and postnatal care. Obstetricians who attended prenatal care were referred to in this paper as “prenatal care doctor”. In this paper, healthcare providers were referred to prenatal care doctors and delivery obstetricians. An estimated 90% of the women who gave birth in this hospital attended a postnatal check-up 42–56 days after the delivery. In 2006, women could freely choose and change the places of maternal care. Prenatal and postnatal check-ups were free, but women had to pay for tests and drugs. A fee was paid for deliveries, and the costs for CS were more than for vaginal delivery. According to our knowledge, some women would discuss with healthcare providers on mode of delivery and then made their own decision, while most of women adhere to healthcare providers’ suggestions.

All primiparous women having given birth in the study hospital were eligible. Those women having their first postnatal check-up during the study period were recruited. A researcher (YZ) or trained nurse in the hospital gave a questionnaire to the woman in her first postnatal check-up and explained how to fill the questionnaire, particularly for the open questions. The woman completed the questionnaire and returned it to the researcher/nurse during the same visit. Women who participated in the survey gave informed consent voluntarily. Questionnaires were given an identification number in place of names. Nurses recorded the woman’s identification number and name in a separate list. The name was used to complete the questionnaire data on CS indications using the medical records. The research group obtained permission of the hospital administration and women that nurses could check women’s medical records and fill reasons for CS in the questionnaire.

Out of the 300 women invited, 272 (91%) completed the questionnaire. The main reasons for refusals were that women did not want to give personal information, they perceived the questionnaire as too long, or they did not have time. Of the 272 women, 9 did not report their delivery mode preferences during pregnancy and were excluded, leaving 263 women for analysis.

The questionnaire was designed by the study group to collect information on women’s willingness and actual delivery mode. The questionnaire was pilot-tested to assess the understandability and feasibility. It was finalized after revising according to the pilot results. In this study, the validation of questionnaire was not essential and was not tested. The final questionnaire included three parts which were women’s demographic and early pregnancy information (such as age, occupation, socioeconomic characteristics, the pregnancy history etc.), information during pregnancy (such as use of maternity care, preparatory exercises, delivery mode preferences, suggestion about delivery mode from other people, etc.), information about staying in hospital and delivering (such as actual delivery mode, environment of hospital, delivery model of other woman in ward, etc.). We uploaded the questionnaire that was translated in English as an Additional file 1. During the first postnatal check-up, women recalled their preferred delivery mode in early pregnancy and in late pregnancy. The actual delivery mode was asked with the question: “Delivery mode in this birth was: 1) CS; 2) natural delivery (“ziran fenmian”; referred to in this paper as “vaginal delivery”). If a woman chose CS, she was asked to give the reason for it. If a woman reported having CS for medical reasons, the nurse checked the patient record and confirmed or corrected the medical reasons. In the analysis, CSs were categorized as having CS with medical indications according to a reason confirmed from the medical record or without medical indications based only on women’s self-report. We did not evaluate the accuracy of the medical reason as defined in the medical record.

The explanatory variables relating to CS were: maternal age; education; family monthly income per person; women’s recall of suggestions for CS given by prenatal care doctors and delivery obstetricians; frequency of prenatal visit; attending childbirth education (e.g. nutrition education during pregnancy and psychological support for childbirth etc.); having preparatory exercises for facilitating delivery during pregnancy (e.g. walking or pregnancy gymnastics etc.); staying in a ward in which most women had CS; being in the hospital before delivery more than five days; recall of own confidence in giving birth vaginally before birth.

Pearson *χ*^2^ and the Generalized Estimating Equation (GEE) model for repeated-measure data analysis with unstructured working correlation matrix were used to test the statistical significance of the differences in the recalled delivery mode preference in early and late pregnancy and the actual delivery mode. Logistic regressions were used to study the association between explanatory variables and having CSs. Each explanatory variable was adjusted for maternal age, education and income in the logistic model, respectively. The software SAS 9.2 was used for statistical analyses. The study design was reviewed for ethical consideration by the Institutional Review Board (IRB) of the school of public health, Fudan University.

## Results

### Background characteristics of all women

Most (84%) of the 263 women were aged between 25 and 34 years, 68% had a college or higher level education, and 64% were relatively wealthy (family monthly income per person more than 3000 renminbi (RMB) (see Table 1).

**Table 1:** **Women’s background characteristics and use of prenatal care by delivery mode (%)**

Description	Total	Vaginal delivery	CS ^a^ total	CS without clinical indication
(Number of women)	(263)	(112)	(151)	(104)
Age group				
<25	10.2	9.2	11.0	13.7
25-29	52.9	53.2	52.7	50.0
30-34	30.6	34.9	27.4	28.4
35+	6.3	2.8	8.9	7.8
Education				
Primary school and lower	1.5	0.0	1.3	0.0
Junior middle school	6.5	5.5	7.3	6.8
Senior middle school	23.8	23.9	24.0	28.2
College and higher	68.2	70.6	67.3	65.0
Monthly income ≥ RMB 3000^b^	64.1	64.5	63.9	61.0
No previous pregnancies	83.2	86.1	81.0	84.7
Preparatory exercises^c^	43.4	55.4	34.5**	32.7
Attended childbirth education	68.4	59.8	74.8**	73.1
Prenatal visits ≥8	76.7	78.6	75.2	74.0
In the hospital before delivery >5 days	41.8	39.8	43.4	37.5

^a^CS: cesarean section.

^b^Monthly income: family monthly income per person.

^c^Preparatory exercises: preparatory exercises for facilitating delivery during pregnancy.

**Difference between women having vaginal delivery and cesarean section, p value < 0.01.

Note: Totally missing cases were few: 2–13 per variable.

All women were having their first baby, but 17% had a history of induced abortion and/or stillbirth. Less than half of women reported having preparatory exercises for facilitating delivery during pregnancy. Most women had more than eight prenatal visits, had attended childbirth education, and had stayed in the hospital before delivery for an average of 5 days.

### Women with CS or vaginal delivery

The CS rate was 57% (151/263), with 17% (n = 47) having CS on medical indications and 40% (n = 104) having it without medical indication. Women having CS were similar to women having vaginal delivery in regard to age, education and monthly income (Table 1). They were also similar in regard to previous pregnancies and having had many prenatal visits, but they less often had preparatory exercises for facilitating delivery during pregnancy and they had more often attended childbirth education than women having vaginal delivery (p < 0.01, Table 1).

**Figure 1:**
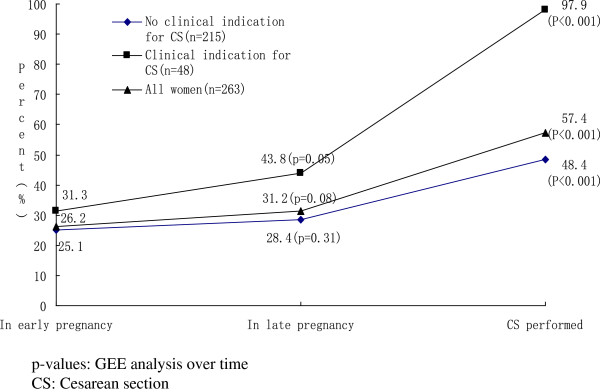
**Women’s preference for cesarean section during pregnancy and actual delivery mode by clinical indication for cesarean section, as reported in the first postnatal visit (%).**

There were 48 women who had a recorded medical indication for CS. The most common indications according to patient records were slow labor progress (n = 6), large-for-date fetus (n = 6), fetal distress (n = 8), uterine scar (n = 4), pregnancy induced hypertension (n = 4), hydramnios (n = 4) and knot in umbilical cord (n = 4). Of the 48 women, 31% reported a preference for CS in early pregnancy and 44% in late pregnancy (p = 0.05); 47 of the 48 women with a medical indication for CS actually had a CS (p < 0.01) (Figure 1).

### CS without medical indications

Of the women without a medical indication for CS (n = 215), 48% had a CS. Of them, 87% had expressed a delivery mode preference in early pregnancy, with 25% wishing for CS and 62% for vaginal delivery. In late pregnancy, 89% of women recalled a delivery mode preference, and 28% recalled it to be CS. The difference in preferences for CS between early and late pregnancy was not statistically significant (Figure 1), with some women (n = 19) changing from a preference for vaginal delivery to one for CS and others (n = 16) having no preference for CS in late pregnancy in spite of a preference for CS in early pregnancy. There was a large difference between preferences for CS and the actual delivery mode employed: 28% of the 131 women who had a preference for a vaginal delivery and 70% of the 23 women who had no delivery mode preference in late pregnancy had CS. Of the 61 women who had a preference in late pregnancy for CS, 85% had had CS (Table 2).

**Table 2:** **Preference for and actual delivery mode (n = 215), women without clinical indication for cesarean section, numbers of women (%)**

		Preference in late pregnancy			
		Vaginal	CS	No preference	Total
Preference in early pregnancy	Vaginal	103(47.9)	19(8.8)	12(5.6)	134(62.3)
	CS	16(7.4)	34(15.8)	4(1.9)	54(25.1)
	No preference	12(5.6)	8(3.7)	7(3.2)	27(12.6)
	Total	131(60.9)	61(28.4)	23(10.7)	215(100.0)
Actual mode of delivery	Vaginal	95(44.2)	9(4.2)	7(3.3)	111(51.6)
	CS	36(16.7)	52(24.2)	16(7.4)	104(48.4)
	Total	131(60.9)	61(28.4)	23(10.7)	215(100.0)

CS: cesarean section.

The women who did not have a medical indication for CS and recalled a delivery mode preference in late pregnancy (n = 192) were grouped into three categories: 1) preferring vaginal delivery but having CS; 2) preferring and having CS; 3) preferring and having vaginal delivery (Table 3). The 9 women with a preference in late pregnancy for CS but having vaginal delivery were excluded from these analyses.

**Table 3:** **Characteristics of women preferring vaginal but having had cesarean section (vaginal/cesarean section), preferring and having had cesarean section (cesarean section/cesarean section) compared to women preferring and having had vaginal delivery (vaginal/vaginal), percentages, crude and adjusted odds ratios** ^**a**^ **(OR, 95% confidence intervals CI), women without clinical indication for cesarean section (n = 192)**

	Vaginal/CS ^b^ (A)	CS/CS (B)	Vaginal/vaginal (C)	(A) vs. (C)	(A) vs. (C)	(B) vs. (C)	(B) vs. (C)
	%	%	%	Crude OR (95% CI)	Adjusted OR (95% CI)	Crude OR (95% CI)	Adjusted OR (95% CI)
(number of women)	(36)	(52)	(95)				
Age ≥30	23.5	46.2	41.3	0.44 (0.18-1.07)	--	1.22 (0.61-2.42)	--
College and higher	60.0	69.2	68.1	0.70 (0.31-1.57)	--	1.05 (0.52-2.19)	--
Monthly income ≥ RMB 3000^c^	52.9	66.0	67.8	0.53 (0.24-1.20)	--	0.92 (0.44-1.92)	--
Preparatory exercises^d^	58.8	18.0	62.6	0.85 (0.38-1.90)	0.97 (0.40-2.33)	0.13 (0.06-0.30)	0.11 (0.04-0.26)^*^
Attended childbirth education	80.6	75.0	60.0	2.76 (1.10-6.94)	3.35 (1.23-9.13)^*^	2.00 (0.94-4.23)	2.59 (1.16-5.79)^*^
Prenatal visits ≥8	75.0	75.5	77.9	0.85 (0.35-2.09)	1.29 (0.47-3.59)	0.87 (0.39-1.97)	0.91 (0.40-2.10)
In the hospital before delivery > 5 days	53.1	30.2	38.8	1.79 (0.79-4.05)	1.34 (0.56-3.20)	0.68 (0.31-1.49)	0.66 (0.29-1.50)
Most women in the same/neighboring wards having CS	58.3	44.2	30.5	3.19 (1.44-7.05)	4.28 (1.71-10.7)^*^	1.80 (0.90-3.64)	2.05 (0.97-4.33)
Confidence in vaginal delivery	63.9	70.8	94.7	0.10 (0.03-0.31)	0.11 (0.03-0.34)^*^	0.14 (0.05-0.41)	0.14 (0.05-0.42)^*^
Prenatal doctors suggestion for CS	30.6	59.6	2.1	20.46 (4.26-98.34)	20.40 (3.88-107.10)^*^	68.64 (15.22-309.57)	86.01 (17.16-431.14)^*^
Delivery obstetricians suggestion for CS	44.4	11.5	4.2	18.20 (5.49-60.29)	25.73 (6.26-105.82)^*^	2.97 (0.80-11.04)	4.32 (0.98-19.05)

^a^Adjusted for age, education and income.

^b^CS: cesarean section.

^c^Monthly income: family monthly income per person.

^d^Preparatory exercises: preparatory exercises for facilitating delivery during pregnancy.

*P value <0.05.

Compared to women preferring and having vaginal delivery, women preferring vaginal delivery but having CS were younger and less often had a higher education or high income (see Table 3). They were similar in regard to having had preparatory exercises during pregnancy for facilitating delivery and having had many prenatal visits, but more of those having had CS reported having attended childbirth education, having staying over five days in the hospital before delivery, having staying in a ward with many women having CS, and reporting less confidence in vaginal delivery before delivery. The differences between the groups in regard to suggestions for CS from their prenatal care doctors and delivery obstetricians were large. After adjusting for maternal age, education and income, women who preferred vaginal delivery but had had CS were 20 times more likely to have received a suggestion for CS from a prenatal doctor and 26 times more likely to have received a suggestion for CS from an obstetrician (Table 3). The differences for the other explanatory variables reported above remained statistically significant by groups with the exception of long stay in the hospital before delivery (see Table 3).

Women preferring and having CS were similar to women preferring and having vaginal delivery in regard to age, education, income and having had many prenatal visits and having a long stay in the hospital before delivery (Table 3), though they were much less likely to have had preparatory exercises during pregnancy for facilitating delivery, were more likely to have attended childbirth education and having stayed in a ward with many women having CS, and they less frequently reported having had confidence in vaginal delivery. But among women who had preferred and had had CS, prenatal care doctors had much more often recommended CS than among women preferring and having had vaginal delivery [adjusted odds ratios (95% confidence interval): 86 (17.2-431.1)].

## Discussion

We found that overall the CS rate was high among women without medical indications for CS. There was a slight increase between early pregnancy and late pregnancy in women’s recalled preference for CS; more women gave birth by CS than had expressed a preference for it. Among women recalled preferring vaginal delivery but having had CS, the suggestions from healthcare providers during pregnancy and labor for CS (as reported by women) were important in women’s decision-making. Among women recalled preferring and having CS, having received a recommendation for CS from the prenatal care doctor was very common. In addition, attending childbirth education and staying in a ward with many women who had had CS were also associated with having CSs.

There are several limitations to this study. Firstly, women’s reported delivery mode preference during pregnancy may not be accurate or are subject to recall bias, as all information was collected post-delivery. Secondly, we included women having given birth and having their first postnatal check-up in the study hospital. We do not know the characteristics of women who gave birth in the hospital but did not attend the same hospital for their postnatal check-up. As around 90% of women had made postnatal check-up in this hospital in 2006, selection bias is unlikely. Thirdly, there can be some bias in classifying CSs into those with and without clinical indications. Women were given the instruction of filling the questionnaire, particularly response to reasons for CS before the survey. But there might be report bias and only those that women reported as being for medical reasons were checked against the hospital records. The odds ratios for healthcare providers’ recommendations for CS were high and the confidence intervals were broad. They may indicate a possible random error and do not necessarily indicate causality. In addition, data of this study were collected in one university affiliated obstetrics and gynecology hospital in 2006–2007. The interpretation of findings was based on our knowledge and referred to recent studies of CS conducted in China, but generalizations should be made with caution.

Based on the published data, this study is the first time that women’s delivery mode preference has been asked and compared to actual delivery mode in (mainland) China; likewise, the associated factors were analyzed with a reasonable sample size. The findings of this study have important research and policy implications for controlling the high CS rate in China, as they suggest healthcare providers have the crucial role in the epidemic.

The overall CS rate in our study (57%) was similar to results from 24 hospitals (58%) that took part in a nationwide survey in 2005–2006 [22]. In this study, 48 women had a recorded medical indication for CS and almost all of them (47 out of 48) had a necessary CS suggesting well coping with the needs. However, we found that more CSs were not medically indicated in this study. Over the past three decades, living standards in China have greatly improved with a rising demand for health. Medical technology has spread and become especially concentrated in big cities. Shifting from an estimated low rate of CSs (3.4% in 1988) to overuse (39% in 2008) has been a public health concern in China, both due to safety and the quality of care, as well as the impacts on efficiency in health services delivery [9, 21, 23].

Women’s preference for CS has been presented as an important reason for the increase in the CS rate [24]. A systematic review, however, concluded that only a few women across a wide variety of countries prefer CS [25]. Many studies in China have reported that fear of pain and perceived better health for the child and mother are the main reasons for women requesting CS [17–19]. Nevertheless, pressure from patients may also reflect the opinions of their healthcare provider on CS. We found that 60% of women who recalled a preference for CS and had CS had received suggestions for a CS delivery from prenatal care doctors, while around half of women who recalled a preference for vaginal delivery but had CS had received suggestions for a CS delivery mode from delivery obstetricians. Women who had received childbirth education were more likely to have had CS. A contrary result was reported in a longitudinal observational study in Hong Kong: many women changed from preferring elective CS to preferring vaginal delivery following an increase in women’s knowledge and information about childbirth [26]. The CS rate was 13% in the Hong Kong study. Studies in Finland and Norway have found that appropriate counseling and psychological therapy as well as obstetric and midwifery support were associated with lower birth concerns and fewer requests for CS [27, 28]. All these studies support our findings of the significance of healthcare providers’ role in women’s choice of delivery mode.

In China, it has been argued that the fee-for-service payment model combined with a bonus system linking a healthcare provider’s salary have pushed healthcare providers to pursue profitable health services [29]. In urban China in 2005, the average expense of CS for a woman ranged from USD 600 to USD 1000 [30], which was 3–4 times more expensive than vaginal delivery. Healthcare providers prefer CS [9, 21] probably because of its high profits. As childbirth is an important event in the family, especially for the first-time parent, women usually comply with healthcare providers’ suggestions in spite of the cost of the services. Equalization in access to high-quality maternity care has been a health priority in China. If the preference among women for CS is due to profit-driven healthcare provider practices or a lack of standardized practice guidelines, then efforts to reduce unnecessary CSs should focus on hospital management, including optimizing the payment methods to providers and supervising health care provider’s performance.

In addition, the hospital environment also influences women’s decision-making on delivery mode. Our study found that women stayed in hospital on average for 5 days prior to delivery. We do not have an explanation for this long stay in the hospital before the delivery. It may be that women have assumed the delivery will take place on the expected day and they have attended hospital in the absence of contractions, and the hospital has then let them stay; or it may be a safety consideration, avoiding an emergency situation while in transit to the hospital. Women who recalled a preference for vaginal delivery but who had had CS were more likely to have more days in the hospital before delivery than women recalled preferring and having vaginal delivery, although the difference was not statistically significant. During the prenatal stay, women saw women who had had CS and this may raise anxiety about their own birth, as they reported having had less confidence in vaginal delivery. Women were more likely to change their delivery mode preference when they stayed in a ward that had many CSs.

## Conclusion

In the primiparous women without a medical indication for CS, women recall of a provider suggestion for CS was a strong predictor of CS both among women who recalled a preference for CS and among women who recalled a preference for vaginal delivery. In addition, attending childbirth education and staying in a ward with many women who had had CS were also associated with having CSs.

The results implied that healthcare providers have the crucial role in controlling the high CS rate in China. Public health education needs strengthening, including discussion of the risks associated with CS and psychological and social support given to women to help them prepare for and cope with childbirth.

## Electronic supplementary material

Additional file 1: **Investigation Questionnaire of Preference Delivery mode of the Primiparous Women.** (DOCX 28 KB)
