# Supplementary material for: Cesarean section in Shanghai: women’s or healthcare provider’s preferences?
Source: BMC Pregnancy Childbirth. 2014 Aug 22;14:285. doi: 10.1186/1471-2393-14-285 (PMC4148545; doi:10.1186/1471-2393-14-285)
Supplement: Supplementary file 1 — Additional file 1: Investigation Questionnaire of Preference Delivery mode of the Primiparous Women. (DOCX 28 KB) [file 12884_2013_1156_MOESM1_ESM.docx]

Investigation Date：______ /___/___(Y/M/D)

ID：_______ Initial of Name _ _ _

**Investigation Questionnaire of Preference Delivery mode of the Primiparous Women**

**Part I**

1. **Demographic**

A1. Date of Birth ：_____/____/____(Y/M/D)

A2. High：_____CM Weight：______kg

A3. Your highest education level is_____:

① illiterate;② primary school; ③ junior high school; ④ senior high school; ⑤ college and above.

A4. Your monthly family income(per person) is_____:

① less than RMB 1000; ②RMB 1000~2000; ③RMB 2000~3000; ④RMB 3000 and above.

A5. Your occupation is ________________

A6. Expected Date of Confinement :______/___/___(Y/M/D)

A7. Pregnancy history:

Number of pregnancies___ Number of abortions___

Number of induced abortions___ Number of live births___ Number of stillbirths___

A8. Did you suffer serious diseases in the past _____?

① Yes, description___________ ②No

1. **Preferred delivery model**

B1. What was the delivery mode you preferred in early pregnancy? _____

① It must be cesarean section; ② It may be cesarean section; ③ Both were okay (depending on the situation during the delivery); ④ I preferred to give birth by myself; ⑤ It must be giving birth by myself if there was no emergency situation; ⑥ I don't know.

B2. What was the primary reason of your choosing on delivery model ? _____

① It was cheap ② I would take less pain ③ Delivery process was shorter ④ I could recover quickly ⑤ It's good for baby ⑥ I could keep good figure ⑦ It was safe ⑧ It was not so hard to myself ⑨ Nearly every women had chosen that ⑩ others（description _____________________________________）

**Part II**

Memo: Questions in this part are asking about information in late pregnancy which is before being in hospital for delivery.

1. **Preferred delivery model**

C1. What was the delivery mode you preferred in late pregnancy?_____

① It must be cesarean section; ② It may be cesarean section; ③ Both were okay (depending on the situation during the delivery); ④I preferred to give birth by myself; ⑤It must be giving birth by myself if there was no emergency situation; ⑥ I don't know.

C2. What was the primary reason of your choice of delivery model? _____

① It's my own choice ② It's my husband suggestion ③ It's the elder's suggestion ④ It's the suggestion of friends and relations ⑤ It's the doctor's suggestion ⑥ It's the suggestion on books and TVs ⑦ Many women had taken it ⑧ other（description _____________________________________）

1. **Prenatal care**

D1. the score of prenatal check-up

The first time : Grade ____ Score_____

The second time : Grade ____ Score_____

The third time : Grade ____ Score_____

D2. status of fetus

Position of the fetus______ Fetal size : ①small ②normal ③big

D3. Your health status in late pregnancy was _____

① very good ②good ③ not so bad ④ not so good ⑤ a little bad

D4. Your prenatal care doctor(s)’ suggestion on delivery mode was :_____

① I could giving birth by myself; ② I had better to have cesarean section; ③ It's hard to judge; ④ No advice

D5. The reason that prenatal care doctor gave such suggestion were: _____ (multiple choice, listed according to importance)

①it's cheap ②It's Safe ③ It's based on the health situation of pregnant women ④ It's based on the situation of fetus ⑤ It's good for baby ⑥It's good for pregnant women ⑦ Delivery process was shorter ⑧others（description __________________________）

D6. Which delivery mode was your husband preference ____

①cesarean section ② giving birth by myself ③ Both were OK ④no advice

D7. The primary reasons of your husband's choice was _____

①it's cheap ②It's less pain ③ Delivery process is shorter ④ The recovery of women is fast ⑤ It's good for baby ⑥ Woman can keep her figure after delivery ⑦ It's safe ⑧ women won't fell too hard ⑨ Nearly every women had chosen that ⑩others（description __________________________）

D8. Which delivery mode was your elder of family preference ____

①cesarean section ② giving birth by myself ③ Both were OK ④No advice

D9. The primary reasons of elder 's choice is _____

①it's cheap ②It's less pain ③ Delivery process is shorter ④ The recovery of women is fast ⑤ It's good for baby ⑥ Woman can keep her figure after delivery ⑦ It's safe ⑧ women won't fell too hard ⑨ Nearly every women had chosen that ⑩others（description __________________________）

D10. Do you had sisters had been given birth ? ____

①yes ② No

D11. The delivery modes their taking were _____

①most were cesarean section ②most were give birth by themselves ③ It's half to half ④I don't know

D12. Which delivery mode was they suggested ____

①cesarean section ② giving birth by myself ③ Both were OK ④No advice

D13. The primary reasons of their suggestion is _____

①it's cheap ②It's less pain ③ Delivery process is shorter ④ The recovery of women is fast ⑤ It's good for baby ⑥ Woman can keep her figure after delivery ⑦ It's safe ⑧ women won't fell too hard ⑨ Nearly every women had chosen that ⑩others（description __________________________）

D14. Do you have friends who had bad experience of delivery? ___

①yes ② No

D15. Her delivery mode is ___

①cesarean section ② giving birth by herself

D16. Do you have friends being doctor? ___

①yes ② No

D17. Which delivery mode was they suggested ____

①cesarean section ② giving birth by myself ③ Both were OK ④No advice

D18. The primary reasons of their suggestion was _____

①it's cheap ②It's less pain ③ Delivery process is shorter ④ The recovery of women is fast ⑤ It's good for baby ⑥ Woman can keep her figure after delivery ⑦ It's safe ⑧ women won't fell too hard ⑨ Nearly every women had chosen that ⑩others（description __________________________）

D19. The other way you got suggestion of delivery mode are ? _____(multiple selection)

① TV ② newspaper ③ book ④others（description __________________________）

D20. Which was better according to the information you've got from these ways? _____

①cesarean section ② giving birth by myself ③ have no idea

D21. Did you and (or) your husband attend pregnancy school (yunfu xuexiao) or similar education? _____

① Yes; ② No.

D22. Did you do special exercise during pregnancy to ensure smooth delivery? ____

①Yes, please give detailed description:___ ② No

D23. Did you have confidence to delivery by yourself? ____

①Yes ② No

D24. Did you think people around you were all kind to you and took care of you during your pregnance than usual? ____

① yes ② similar as usual ④ not

D25. How many prenatal visits you had taken? ____

① 0 ② 1－5 ③ 6－7 ④ 8－10 ⑤more 10

**Part III**

Memo: Questions in this part are asking about information when you were giving birth and after that.

1. **Delivery mode**

E1. The delivery mode you actually took was____:

① cesarean section; ② natural delivery

E2. If it was a cesarean section, the main reason you took it was_____

① It's my original selection ②It's doctor suggested ③ Because of the status of fetus ④ Because of the health status of myself ⑤ There is unexpected events happened during delivery process ⑥ I lost confidence thinking of the long time delivery process ⑦ It's too pain to bear ⑧ I lost self-confidence during the delivery and was feared that the situation would turn to bad if it was continued ⑨others（description __________________________）

E3．Did your family support your decision? _____

① Yes ② No ③ Didn't care

E4. If you took cesarean section，the indications of CS recorded in your medical record were __________________________________

1. **Hospitalization**

F1. The level of your ward was ____

① general ward(3～6 people) ② relaxing ward (2 people) ③ family ward(1 people)

F2. How many days did you stay in the hospital before delivery? ____days

F3. What was your evaluation to the environment of hospital？_____

① very good ② good ③ not too bad ④ not too good

F4. What were the delivery modes of other women in the same or neighboring wards when you stayed in hospital before delivery?

① majority were cesarean section; ②majority were natural delivery; ③ Half were Cesarean section and half were natural delivery

F5. Did your family come to accompany you?____

① Yes, my husband came ② Yes, someone else of my family came ③ No

F6. Did you have confidence in giving birth by yourself? ____

① Yes; ② No.

1. **Delivery**

G1. When did you go in the delivery room for delivery _____?

① morning ② afternoon ③ evening（18:00-21:00） ④ midnight or before dawn（21:00-6:00）

G2. The delivery process persisted for ____ hours

G3. If 0-10 represented from not painful to very painful, in your opinion the pain level during delivery was ____(0－10)

G4. Have you taken any anti-pain measures during delivery process? _____

① Yes，description________ ② No

G5. (for those preferring nature delivery women) During your delivery , when did you change your mind to take cesarean section? _____hours after going into delivery room

G6. The main reason causing your decision was ______________________________

G7. During labor, obstetrician’s suggestion for delivery mode was____

① I could delivery by myself; ② it was better to have cesarean section; ③ I could decide myself.

G8. Did you feel worried or anxious during delivery process? ____

① Yes ②no

G9. If yes, what you were worried about was __________________________________
